# Antileukemic Activity of hsa-miR-203a-5p by Limiting Glutathione Metabolism in Imatinib-Resistant K562 Cells

**DOI:** 10.3390/cimb44120438

**Published:** 2022-12-19

**Authors:** Priyanka Singh, Radheshyam Yadav, Malkhey Verma, Ravindresh Chhabra

**Affiliations:** 1 Department of Biochemistry, School of Basic Sciences, Central University of Punjab, Ghudda 151401, India; 2 School of Biotechnology, Institute of Science, Banaras Hindu University, Varanasi 221005, India

**Keywords:** chronic myeloid leukemia (CML), chemoresistance, GC-MS, metabolomic profiling

## Abstract

Imatinib has been the first and most successful tyrosine kinase inhibitor (TKI) for chronic myeloid leukemia (CML), but many patients develop resistance to it after a satisfactory response. Glutathione (GSH) metabolism is thought to be one of the factors causing the emergence of imatinib resistance. Since hsa-miR-203a-5p was found to downregulate *Bcr-Abl1* oncogene and also a link between this oncogene and GSH metabolism is reported, the present study aimed to investigate whether hsa-miR-203a-5p could overcome imatinib resistance by targeting GSH metabolism in imatinib-resistant CML cells. After the development of imatinib-resistant K562 (IR-K562) cells by gradually exposing K562 (C) cells to increasing doses of imatinib, resistant cells were transfected with hsa-miR-203a-5p (R+203). Thereafter, cell lysates from various K562 cell sets (imatinib-sensitive, imatinib-resistant, and miR-transfected imatinib-resistant K562 cells) were used for GC-MS-based metabolic profiling. L-alanine, 5-oxoproline (also known as pyroglutamic acid), L-glutamic acid, glycine, and phosphoric acid (Pi)—five metabolites from our data, matched with the enumerated 28 metabolites of the MetaboAnalyst 5.0 for the GSH metabolism. All of these metabolites were present in higher concentrations in IR-K562 cells, but intriguingly, they were all reduced in R+203 and equated to imatinib-sensitive K562 cells (C). Concludingly, the identified metabolites associated with GSH metabolism could be used as diagnostic markers.

## 1. Introduction

Chronic myeloid leukemia (CML), the first human malignancy associated with a single acquired genetic defect, is well-known leukemia identified by the Philadelphia (Ph^+^) chromosome containing the *Bcr-Abl1* fused oncogene [1]. This oncogene encodes the BCR-ABL1 oncoprotein, a constitutively active tyrosine kinase that activates multiple signaling pathways, ultimately leading to aberrant proliferation in CML [2,3]. Many tyrosine kinase inhibitors (TKIs) were developed to restrict this kinase, with imatinib being the most effective [4]. Even though imatinib was a highly effective drug for CML treatment, some patients acquired resistance after the initial response [5]. Several initiatives have been launched to resolve this issue, one of them being translational inhibition by microRNAs (miRNAs). Past in vitro and in vivo studies based on CML treatments have also shown that resistance to imatinib therapy can be reversed with miRNA transfection [6,7,8]. To thoroughly investigate how miRNAs regulate tumorigenesis, a metabolomic-based methodology is being incorporated into cancer therapeutics-based studies. Metabolomics is the scientific study of chemical processes involving metabolites, small molecule substrates, and intermediate products of cell metabolism. In the case of cancer, detailed analysis of altered metabolites may yield biomarkers for diagnostic and therapeutic purposes [9,10]. Something similar was observed in the case of CML, where metabolomics is currently the focus of intensive research, a panel of specific metabolites proved effective in monitoring the efficacy of TKI therapy [11,12].

The most abundant antioxidant metabolism found in living organisms is glutathione (GSH) metabolism, which maintains cellular redox homeostasis. The key player in this metabolism is Glutathione (GSH) which maintains adequate cysteine levels, detoxifies xenobiotics, and regulates transcription factors related to redox signaling [13]. This antioxidant metabolite also confers therapeutic resistance to cancer cells, and GSH overexpression eventually promotes tumor progression [14]. This statement is a little perplexing because glutathione normally maintains cell health by regulating cellular homeostasis. However, tumor cells are different because they have highly reduced intracellular environments that are accompanied by significant levels of reduced glutathione (GSH) (intracellular level of GSH is 90–95% of its total concentration), so investigation into GSH metabolism would aid in understanding the role of GSH and its associated metabolites in the development of chemotherapeutic resistance in different tumors [14,15,16]. Not only GSH is responsible for chemoresistance in the GSH metabolism, but amino acids and inorganic compounds involved in this cellular metabolism also play a significant role [17]. These metabolites support the acquisition of anticancer resistance by providing essential building blocks for biosynthetic pathways and maintaining a balanced redox state, as well as by supporting the reduced intrinsic vulnerability of cancer stem cells to antineoplastic therapy [18]. Additionally, chemoresistance-based studies have reported that alterations in the abundance of amino acids and inorganic compounds related to GSH metabolism are frequently associated with resistance to drugs such as bortezomib, sorafenib, and cisplatin [19,20,21,22].

Remarkably, microRNAs (miRNAs) have been discovered to be potent regulators, either negatively or positively, of various cellular metabolisms in a variety of cancers that ultimately affect cell proliferation [23]. In the case of GSH metabolism too, it gets regulated by several miRNAs such as miR-17-92, miR-125b, miR-126, miR-128a, miR-141, miR-200, miR-21, miR-34a, miR-210, miR-122, miR-335, and miR-320 [24,25,26,27,28,29,30,31]. Hence, in the context of the aforementioned investigations, the current study examined whether the expression of hsa-miR-203a-5p, that have previously been shown to overcome imatinib resistance in leukemic cells [32], could reconfigure the altered GSH metabolism of imatinib-resistant K562 cells (R).

## 2. Materials and Methods

### 2.1. Chemicals

RPMI-1640 cell culture media (HiMedia, Thane, India), Fetal bovine serum (FBS) (Gibco, Brough, UK), Antibiotic-Antimycotic (Gibco, Waltham, MA, USA), Imatinib (Sigma-Aldrich, Beijing, China), Phosphate buffer solution (PBS) (Gibco, Waltham, MA, USA), Trypsin (Hi-media, Thane, India), miRNA mimic: hsa-miR-203a-5p (Sigma-Aldrich, St. Louis, MO, USA), Lipofectamine^TM^ Transfection Reagent (Invitrogen, Waltham, MA, USA), Methanol (Fisher Scientific, Mumbai, India), Norvaline (Sigma-Aldrich, St. Louis, MO, USA), Dichloromethane (ThermoFisher Scientific, Scoresby, VIC, Australia), *N*-trimethylsilyl-*N*-methyl trifluoroacetamide (MSTFA) (Sigma-Aldrich, St. Louis, MO, USA). All compounds were of analytical grade and thus diluted in ultrapure water unless otherwise specified.

### 2.2. Cell Culture

The human CML blast crisis cell line K562 (acquired from NCCS Pune, India) was grown in RPMI-1640 culture medium supplemented with 1% penicillin-streptomycin and 10% FBS and was kept in an incubator (at 37 °C under 5% CO_2_). When sufficient numbers of cells were developed, some of the cells subsequently developed into imatinib-resistant K562 (IR-K562) cells, as described in a previously published study from our laboratory [33]. After successfully establishing IR-K562 cells, hsa-miR-203a-5p was transfected into imatinib-resistant K562 (IR-K562/R) cells using Lipofectamine transfection reagent according to the protocol outlined by the manufacturer. The cells were harvested 24 h post-transfection. The following three sample sets were utilized for the metabolic profiling: (1) K562 cells (C), (2) IR-K562 cells (R), and (3) IR-K562 cells transfected with hsa-miR-203a-5p (R + miR-203).

### 2.3. Metabolite Extraction

All K562 cell lines were centrifuged, and the culture media was disposed of. After that, the cells were gently washed twice with PBS before being resuspended in ice-cold methanol. The supernatants of different cell sets were separated in a fresh falcon as it contained the metabolite extract after centrifugation at 3000× *g* at 4 °C for 10 min. The falcons were stored at −80 °C until further analysis.

### 2.4. Preparation of Samples for GC-MS Analysis

The sample preparation for GC-MS analysis was carried out as explained in previous studies [34,35]. Stored −80 °C falcons were taken out and thawed at room temperature, after which norvaline (1 mg/mL) was added as an internal standard to methanol containing cellular extracts of C, R, and R + miR-203; and the entire solution was transferred to glass vials. The glass vial solutions were then gently dried in an Eppendorf Vacufuge^®^ plus Vacuum Concentrator with a gentle stream of nitrogen gas.

Subsequently, the derivatization process was carried out to volatilize and polarize metabolites such as carbohydrates, steroids, amino acids, and fatty acids [36]. Fifty microliters of dichloromethane (DCM) plus 50 μL of MSTFA (derivatization reagent) was added to the residue left after evaporation in the vacuum concentrator during the derivatization process. The vials were then vortexed and heated at 80 °C for 30 min. Then, 50 μL of the cooled derivatized solutions containing metabolite extracts from different sets of K562 cells was transferred into screw-top autosampler glass vials for GC-MS analysis.

### 2.5. Instrumentation and Chromatographic Parameters

The metabolic profile of all samples (C, R, and R + miR-203) was obtained using GCMS-QP2010 Ultra (Shimadzu) at the Central Instrumentation Laboratory (CIL), Central University of Punjab, Bathinda. The column temperature was set to 70 °C for 2 min, then ramped at 15 °C/min to 250 °C, held for 2 min, and at last, raised by 10 °C/min to 300 °C, then withstood for 8 min, for a complete run time of 29 min. The samples (1 μL) were introduced in the instrument in the split mode of a 10:1 ratio with the injector temperature set at 250 °C and the flux rate of the carrier gas, helium, as 1 mL/min. The temperature of the transfer line was 250 °C, that of the manifold was 40 °C and the ion source had a temperature of 200 °C.

The electron multiplier was set to relative mode with auto-tune, and the ionization was accomplished with a 1.02 kV electron beam. Electron Impact (EI) mode was active on the MS detector. The mass spectra were recorded at a rate of six scans per second over a range of 50–1000 *m*/*z*. The intensity and resolution of chromatographic peaks in all samples were investigated using norvaline as an internal standard.

### 2.6. Data Acquisition for GC-MS

Data files from GC-TQ-MS were extracted as Computable Document Format (CDF) files. To make the instrumental data set more comprehensible for data analysis, any biases such as background noise and retention time (RT) fluctuations were eliminated to make the instrumental data set more understandable for data analysis. For data pre-processing, MZmine 2.21 software was used [37]. The parameters were chosen for this procedure as: RT range 3.0–29.0 min; *m*/*z* range 50–600; MS data noise level 2.5 × 10^4^; *m*/*z* tolerance 0.5; chromatogram baseline level 2.0 × 10^4^; peak duration range 0.05–0.50 min.

### 2.7. Identification of Metabolites

The National Institute of Standards and Technology Mass Spectral Library (NIST17M1.Lib) database was used for the identification of specific compounds. In addition, the name, molecular weight, and CAS number of each compound were ascertained by NIST WebBook and PubChem.

When metabolites were identified, their area under curve (AUC) values were calculated and the data were normalized by dividing the area of each metabolite by the area of norvaline (internal standard). Eventually, the normalized AUC values were considered as the concentration of each metabolite; and metabolic profiling and statistical analysis of each sample (C, R, and R + miR-203) were performed afterward. SMPDB (Small Molecule Pathway Database), Human Metabolic Database (HMDB), and Kyoto Encyclopedia of Genes and Genomes were used to obtain data for pathways and metabolites (KEGG).

### 2.8. Analysis of Metabolic Pathways

Metabolite Set Enrichment Analysis (MSEA), notably pathway over-representation analysis (ORA), was executed on the GC-MS metabolite data using the free online software MetaboAnalyst 5.0 [38]. This was used to identify biologically significant patterns in resistant cells and to verify which metabolic pathways were altered and contributed the most to the distinction between different K562 cell sets (C, R, and R + miR-203).

### 2.9. Statistical Analysis

Following enrichment analysis, the retrieved data was statistically analyzed using GraphPad Prism version 5 (GraphPad Software, San Diego, CA, USA).

## 3. Results

### 3.1. Target Identification of hsa-miR-203a-5p

**Figure 1:**
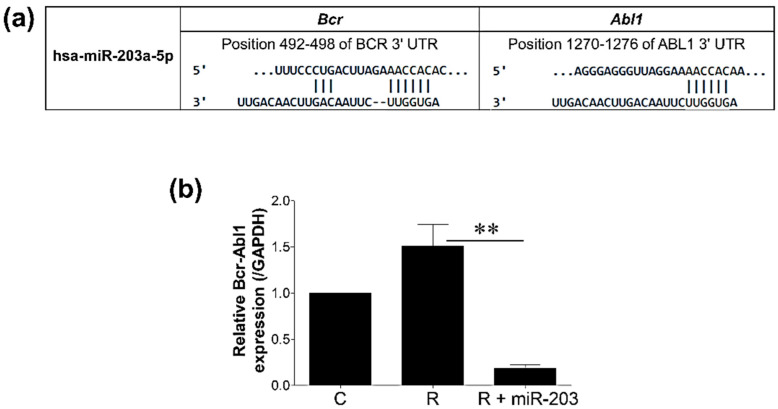
(**a**) Schematic illustration of predicted target regions of hsa-miR-203a-5p in 3′UTR of Bcr and Abl1 subunits of the Bcr-Abl1 oncogene using TargetScan (**b**) Relative gene expression of Bcr-Abl1 (/GAPDH) following miRNA transfection. C: imatinib-sensitive K562 cells R: imatinib-resistant K562 cells (IR-K562 cells), R + miR-203: IR-K562 transfected with hsa-miR-203a-5p. Data are presented as mean ± SD (n = 3) with statistical significance ** *p* ≤ 0.005.

MiRNAs are frequently implicated in cancer chemoresistance, and hsa-miR-203a-5p has been extensively studied in vitro and in vivo to overcome imatinib resistance [32,39,40]. As evidenced by TargetScan, this could be due to the presence of hsa-miR-203a-5p binding sites in both the *Bcr* and *Abl1* subunits of the *Bcr-Abl1* oncogene (Figure 1a). Furthermore, qRT-PCR results revealed that hsa-miR-203a-5p significantly suppressed *Bcr-Abl1* expression, as endogenous expression was low (0.09-fold) in miR-transfected cells compared to resistant cells (Figure 1b).

BCR-ABL1-positive cells were also reported to have altered cellular metabolisms, which were involved in the development of imatinib resistance [41,42,43]. Correspondingly, since hsa-miR-203a-5p was found to downregulate *Bcr-Abl1* oncogene, we postulated that ectopic expression of this miRNA could make resistant cells more susceptible to imatinib by restoring the altered metabolomics of imatinib-resistant K562 cells.

### 3.2. Pathway Enrichment Analysis Utilizing Metabolite Sets in Imatinib-Sensitive (C) and -Resistant (R) K562 Cells

**Figure 2:**
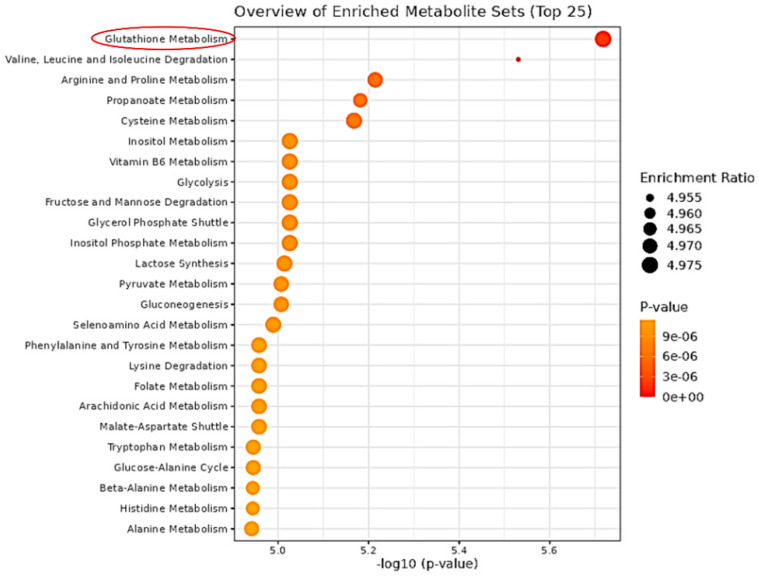
Overview of metabolite sets enrichment: Quantitative Enrichment Analysis (QEA) algorithm summary plot for all significantly transformed metabolites between the imatinib-sensitive (C) and imatinib-resistant (R) K562 cells.

To see the effect of resistance and its reversibility after miRNA transfection, the intracellular GC-MS-based metabolic profiling of K562 cells (C), IR-K562 cells (R), and hsa-miR-203a-5p transfected IR-K562 cells (R + miR-203) were carried out. GC-MS profiling identified 75 major metabolites in total, 32 of which were reported in all sets of K562 cells. These common metabolites were subjected to the MetaboAnalyst 5.0 metabolite set enrichment analysis (MSEA) to identify biologically relevant patterns that are significantly enriched in quantitative metabolomic data. MSEA offers three enrichment analysis algorithms, and for our analysis, we chose the Quantitative Enrichment Analysis (QEA) algorithm. QEA requires a compound concentration table—entered as a comma-separated (.csv) file with each sample per row and each metabolite concentration per column. The analysis revealed that GSH metabolism is the most enriched cellular metabolism in imatinib-sensitive (C) versus -resistant (R) K562 cells, and the same cellular metabolism may be responsible for resistance emergence in imatinib-resistant K562 cells (Figure 2). The conclusion that GSH metabolism was possibly responsible for the emergence of resistance was supported by earlier research, which revealed that drug-sensitive CML cells had lower glutathione levels than resistant cells [44,45].

**Figure 3:**
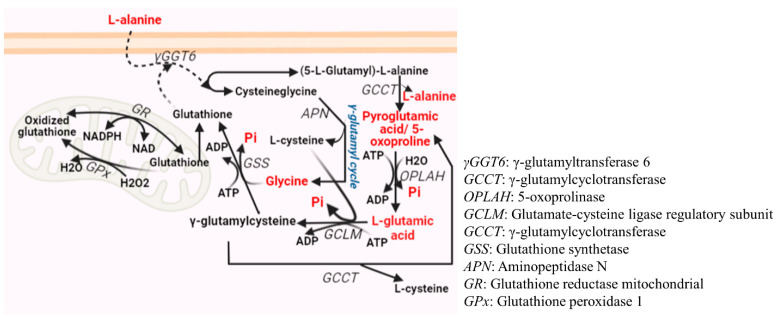
Glutathione (GSH) Metabolism Overview: L-glutamic acid, cysteine, and glycine sequentially synthesize GSH by glutamylcysteine synthetase and GSH synthetase, respectively. L-glutamic acid and cysteine are combined by glutamate-cysteine ligase, which is powered by ATP, to form γ-glutamylcysteine. The enzyme glutathione synthetase, which is also powered by ATP, can synthesize γ-glutamylcysteine and glycine. GSH exists in two states: oxidized (GSSG) and reduced (GSH). GSH oxidation occurs as a result of a relatively high GSH concentration within cells. Figure created with BioRender.com. (Note: Metabolites—L-alanine, 5-oxoproline/pyroglutamic acid, L-glutamic acid, glycine, and phosphoric acid (Pi)—matching the user-uploaded data with the 28 metabolites listed by MetaboAnalyst are indicated in red text).

Then, by uploading the list of GC-MS-based identified metabolites, the pathway analysis feature of MetaboAnalyst was exploited, and it was determined that five (depicted in red text in Figure 3) metabolites for GSH metabolism—L-alanine, 5-oxoproline (also known as pyroglutamic acid), L-glutamic acid, glycine, and phosphoric acid (Pi)—matched with our data with the enlisted 28 metabolites of the MetaboAnalyst. Of the five identified metabolites, L-glutamic acid and glycine are the most important as they both are precursor amino acids of GSH [13].

### 3.3. Comparison of Glutathione (GSH) Metabolism-Related Metabolites in Different K562 Cell Sets

**Figure 4:**
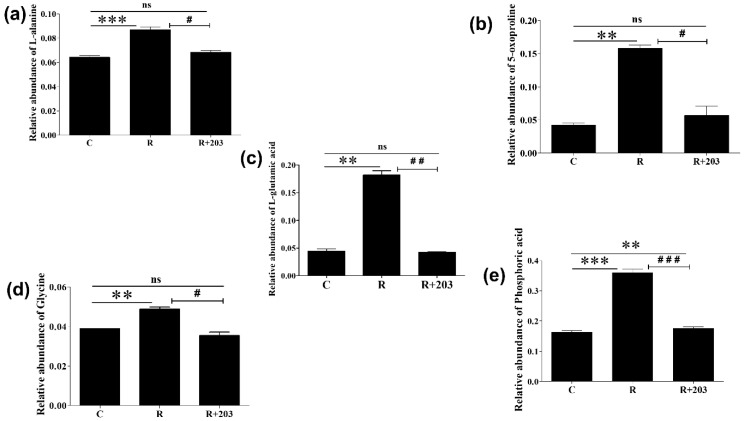
Bar plot showing the relative abundance of—(**a**) L-alanine, (**b**) 5-oxoproline (also known as pyroglutamic acid), (**c**) L-glutamic acid, (**d**) glycine, and (**e**) phosphoric acid (Pi)—the metabolites involved in glutathione (GSH) metabolism. All data are presented as mean ± SD (*n* = 3) with statistical significance ^#^ *p* ≤ 0.05, **, ^##^ *p* ≤ 0.005, and ***, ^###^ *p* ≤ 0.0005.(ns: non-significant, C: imatinib-sensitive K562 cells, R: imatinib-resistant K562 cells, R + 203: hsa-miR-203a-5p transfected imatinib-resistant K562 cells).

Following the revelation of glutathione (GSH) metabolism and five metabolites (L-alanine, 5-oxoproline, L-glutamic acid, glycine, and phosphoric acid) that are involved in this metabolism, the relative abundance of these selected metabolites was investigated in subsequent studies after hsa-miR-203a-5p transfection to investigate whether the miRNA could overcome resistance by reducing the levels of these metabolites (Figure 4).

All five metabolites were found in higher concentrations in imatinib-resistant (R) than in imatinib-sensitive (C) K562 cells, but their relative abundance was significantly reduced when resistant cells were transfected with hsa-miR-203a-5p (R + 203) (Figure 4). Except for the phosphoric acid, there was no statistically significant (ns) difference in relative abundance between sensitive (C) and miRNA-transfected (R + 203) K562 cells which means that transfected cells exhibited levels of L-alanine, 5-oxoproline, L-glutamic acid, and phosphoric acid approximately equivalent to imatinib-sensitive K562 cells.

Levels of L-alanine, the metabolite that forms cysteineglycine and (5-L-glutamyl)-L-alanine with GSH, were found to be significantly higher (1.35-fold) in imatinib-resistant (R) than imatinib-sensitive (C) K562 cells, and it declined by 0.79-fold in hsa-miR-203a-5p transfected resistant K562 cells (R + 203) (Figure 4a). Likewise, the 5-oxoproline level was relatively high in R cells than in C cells (3.75-fold), and this decreased significantly in the presence of miRNA (0.36-fold) (Figure 4b). L-glutamic acid, a key metabolite of GSH metabolism, increased significantly (4.12-fold) in resistant cells, whereas R+203 showed a substantial decrease (0.23-fold) in contrast to resistant cells (Figure 4c). Glycine, an important amino acid that is essential for GSH production, increased by 1.29-fold in resistant cells compared with imatinib-sensitive K562 cells and decreased by 0.73-fold in miRNA-transfected resistant cells (Figure 4d). Once resistant K562 cells were transfected with hsa-miR-203a-5p, the fold change for phosphoric acid (Pi), a notable inorganic compound of GSH metabolism, considerably decreased (0.49-fold) compared to imatinib-resistant cells (Figure 4e).

## 4. Discussion

Despite significant therapeutic advances, drug resistance remains the primary obstacle to effective cancer treatment, and the same holds for imatinib in the case of CML patients. Many factors contribute to drug resistance, one of which is cellular metabolism, which is generally beneficial to normal cells but causes tumorigenesis in cancer cells after being altered by overstimulated growth factors. This altered metabolism along with mitochondrial dysfunction, and persistent genetic alterations in tumor cells causes a high yield of reactive oxygen species (ROS), resulting in the accumulation of oxidized proteins, DNA, and lipids [46]. As a result, cancer cells have elevated levels of ROS-scavenging molecules as an adaptive response, with glutathione (GSH) being the most effective free radical scavenger. This scavenging molecule maintains a highly reducing milieu in the cellular compartments (cytosol, nucleus, mitochondrial matrix, and peroxisome) to facilitate proper folding and activity; furthermore, GSH prevents apoptosis by reacting with ROS in mitochondria [14]. Other than this, high GSH levels in cancer cells also incentivize metastasis and contribute to the emergence of chemotherapy drug resistance [47,48]. Since miRNAs play a role in the regulation of cellular metabolism by directly targeting enzymes or indirectly by modulating the expression of transcription factors [49,50], a miRNA-based approach to restrain metabolisms may be expected to overcome imatinib resistance.

Taking into account all of these facts, the current study was conducted in the anticipation that hsa-miR-203a-5p would reduce GSH metabolism to overcome imatinib resistance, as the GC-MS results demonstrated that GSH metabolism is prominent in imatinib-resistant leukemic (IR-K562/R) cells. L-alanine, 5-oxoproline/pyroglutamic acid, L-glutamic acid, glycine, and phosphoric acid (Pi), which were identified through MetaboAnalyst 5.0 to be related to GSH metabolism, were found to be differentially expressed among different K562 cell sets (Figure 4). L-alanine, the metabolite that enters the cell and initiates GSH metabolism, has been recognized as a new cancer-associated biomarker because of its elevated levels in malignant cells [51,52]. Since L-alanine is a glucogenic amino acid, it contributes to gluconeogenesis and meets the immediate energy requirements of cancer cells whenever its levels increase in cancer patients [53,54]. Higher concentrations of pyroglutamic acid, also known as 5-oxoproline, signal upregulated GSH metabolism and oxidative stress [55]. Oxidative stress, which means ROS generation, has long been involved in cancer as tumor cells use ROS to activate protumorigenic signaling processes that support cancer cell growth, survival, angiogenesis, and metastasis [56,57]. Adenosine triphosphate (ATP), the driving force behind all metabolisms, leads to the generation of phosphoric acid (Pi) while regulating the autophosphorylation of signaling pathway components [58]. Phosphoric acid (Pi) maintains an acidic environment in cancer cells, which promotes immunosuppression and unregulated cancer cell proliferation [59]. The increased production of phosphoric acid (Pi) in resistant cells indicated that ATP catabolism occurred more rapidly in resistant cells than in control cells, suggesting that glutathione metabolism was speeding up to meet the reduced milieu demand of resistant leukemic cells.

Since GSH (L-glutamyl-L-cysteinyl-glycine) is a tripeptide that incorporates L-glutamic acid and glycine, the rate of GSH synthesis is influenced by these amino acids and both are well-studied GSH metabolites in the context of cancer [60]. The determined values of L-glutamic acid and glycine in the current study assisted in differentiating the metabolic profiles of imatinib-sensitive, imatinib-resistant, and miRNA-transfected leukemic K562 cells and better understanding the cause of drug resistance. In tumor cells, in addition to participating in GSH metabolism, L-glutamic acid also serves as a nitrogen source in cancer cells alongside L-glutamine [56]. An intriguing fact about L-glutamic acid is that all of its derivatives have the potential to be used therapeutically, and they all behave in a way that is completely different from how L-glutamic acid causes tumorigenesis [55,61]. As a result, L-glutamic acid, its conjugated derivatives, and glutathione metabolism have suddenly become the target of cancer-related research. Glycine, an essential component of GSH, has been identified as a new target for cancer therapeutic interventions as this amino acid contributes to the biosynthesis of purines, and also its catabolism encourages tumorigenesis and malignancy [62]. It has been postulated that glycine availability is the limiting factor in glutathione synthesis [63]. Since cancer cells rely more on L-glutamic acid and glycine, these two glutathione-related metabolites have been identified as possible targets.

Overall, this study uncovered a strong correlation between glutathione metabolism and the emergence of resistance. We can say this with assurance because results are consistent with a previous study by Kalinina et al., which proved that the emergence of cellular drug resistance against doxorubicin (DOX) in erythroleukemic K562 cells was caused by elevated levels of antioxidant enzymes and GSH [64]. Besides that, it was exposed that imatinib resistance could be overcome by lowering the levels of the five aforementioned metabolites through miRNA transfection, as their concentrations in hsa-miR-203a-5p transfected resistant K562 cells (R+203) were equivalent to those in imatinib-sensitive K562 cells (C).

## 5. Conclusions

This study investigated the metabolic profile of different sets of K562 cells and demonstrated that glutathione (GSH) metabolism plays a significant role in tumor initiation, progression, and drug resistance. With a better understanding of this pathway and the role of metabolites related to GSH metabolism, other drugs could be used in combination to effectively limit CML growth. At last, it can be concluded that our result will hopefully lead to the establishment of novel diagnostic tools.

## Data Availability

No datasets were generated for the preparation of this manuscript.
